# Mid-term lifetime survivals of octogenarians following primary and revision total knee arthroplasties were satisfactory: a retrospective single center study in contemporary period

**DOI:** 10.1186/s43019-020-00069-w

**Published:** 2020-09-22

**Authors:** Sang Jun Song, Kang Il Kim, Dae Kyung Bae, Cheol Hee Park

**Affiliations:** 1 Department of Orthopaedic Surgery, College of Medicine, Kyung Hee University, Seoul, South Korea; 2 Department of Orthopaedic Surgery, Seoul Sacred Heart General Hospital, Seoul, South Korea; 3 Department of Medicine, Graduate School, Kyung Hee University, 23 Kyunghee-daero, Dongdaemun-gu, Seoul, 130-872 South Korea

**Keywords:** Octogenarian, Knee, Arthroplasty, Revision, Survival, Complication

## Abstract

**Background:**

As life expectancy increases, the number of octogenarians requiring primary and revision total knee arthroplasty (TKA) is increasing. Recently, primary TKA has become a common treatment option in octogenarians. However, surgeons may still be hesitant about performing revision TKA on octogenarians because of concern about risk and cost benefit. The purpose of this study was to investigate clinical outcomes, postoperative complications, and mid-term lifetime survival in octogenarians after primary and revision TKA.

**Materials and methods:**

We retrospectively reviewed 231 primary TKAs and 41 revision TKAs performed on octogenarians between 2000 and 2016. The mean age of patients undergoing primary TKA was 81.9 years and that of patients undergoing revision TKA was 82.3 years (*p* = 0.310). The age-adjusted Charlson comorbidity index was higher in revision TKA (4.4 vs. 4.8, *p* = 0.003). The Western Ontario and McMaster Universities Osteoarthritis Index (WOMAC) and range of motion (ROM) were evaluated. The incidence of postoperative complications (TKA-related, specific or systemic) and lifetime survival rate (endpoint death determined by telephone or mail communication with patient or family) were investigated.

**Results:**

The WOMAC and ROM improved significantly after primary and revision TKA, although postoperative results were worse in the revision group (33.1 vs. 47.2; 128.9° vs. 113.6°; *p* < 0.001, respectively). There were no cases of aseptic or septic component failure in either group. One case of periprosthetic fracture was observed in the revision group (0% vs. 2.4%, *p* = 0.151), and three cases of deep vein thrombosis (DVT)/pulmonary thromboembolism (PTE) (one case of DVT and two cases of PTE) were observed in the primary group (1.3% vs. 0%, *p* = 1.000). The most common systemic complication in both groups was delirium (7.4% vs. 14.6%, *p* = 0.131). There were no differences between the two groups in the other systemic complication rates. The 5-year and 10-year lifetime survival rates were 87.2% and 62.9%, respectively, in primary TKA and 82.1% and 42.2%, respectively, in revision TKA (*p* = 0.017).

**Conclusions:**

Both primary and revision TKA are viable options for octogenarians, based on the satisfactory clinical outcomes, TKA-related complication rates, and mid-term lifetime survival. Delirium needs to be managed appropriately as the most common systemic complication in both primary and revision TKA in octogenarians.

**Level of evidence:**

IV

## Background

As life expectancy increases, the number of patients outliving their implanted prosthesis is increasing, which will induce an inevitable increase in the need for revision total knee arthroplasty (TKA). Statistical models predict that the number of revision TKAs will increase by 601% by the year 2030 [1, 2]. This trend has also been seen in patients over the age of 80 years. It is expected that the number of revision surgeries in octogenarians will increase fourfold by the year 2050 [1].

Recently, primary TKA has become a common treatment option in octogenarians [3]. The improvements in medical care and high patient satisfaction after surgery justify the use of primary TKA in octogenarians [3, 4]. However, surgeons may still be hesitant about performing revision TKA on octogenarians because of concerns about risks and cost benefit [1]. Compared to primary TKA, revision surgery is technically more demanding, with longer operation times and increased blood loss [5]; such a situation might increase the postoperative risk in vulnerable older patients. Considering additional resources for revision TKA, such as increased implant cost and surgeon’s effort [6], the indexes for evaluation of risk and cost benefit should be investigated when revision surgery is performed in octogenarians.

The purpose of this study was to investigate clinical outcomes, postoperative complications, and mid-term lifetime survival in octogenarians after primary and revision TKA. We hypothesized that the postoperative outcomes in revision TKA are comparable to those in primary TKA in octogenarians.

## Materials and methods

### Patients

This retrospective analysis was conducted using a database at our institution. After reviewing the database, 231 primary TKAs and 41 revision TKAs performed in octogenarians at our institution between January 2000 and December 2016 were identified and included in the study. The cause of primary TKA was degenerative osteoarthritis in 227 cases, rheumatoid arthritis in 3 cases, and osteonecrosis in 1 case. The reason for revision TKA was polyethylene wear and osteolysis in 23 cases, periprosthetic joint infection in 10 cases, component loosening in 4 cases, instability in 2 cases, and periprosthetic fracture in 2 cases. All revision TKAs were performed with exchange of the femoral or tibial components; in the present study there were no cases of simple exchange of the polyethylene insert or revision only of the patellar component. All patients with periprosthetic joint infection were subjected to two-stage revision TKA, and only the second stage of surgery for final implantation was included as a revision procedure. This study was approved by our Institutional Review Board.

### Surgical technique

All primary and revision TKAs were performed by the two senior surgeons. Consistent surgical principles, techniques, and rehabilitation protocols were applied.

The primary TKAs were performed using a modified measured resection technique. A medial parapatellar approach was used. Any contracted medial or lateral soft tissue was selectively released where required. All components were implanted using a total cementation technique. The type of prosthesis was cruciate retaining (CR) in 86 cases, posterior stabilized (PS) in 144 cases, and constrained condylar knee (CCK) in 1 case. The patellae were resurfaced in all cases.

Revision TKAs were performed using the prior midline skin incision using a medial parapatellar approach; no additional supplemental approaches were used. Efforts were made to restore the original joint line via augmentation of the various bone defects and to ensure accurate rotation of an appropriately sized femoral component, without unnecessary release of contracted soft tissue or unnecessary placement of over-thick polyethylene inserts or constrained prostheses [7]. The femoral and tibial components were revised in 34 cases, the femoral component only in 5 cases, and the tibial component only in 2 cases. The long, extended stem was always used with a fully or partially cemented technique. Various surgical strategies were required to manage bone defects. Appropriate thickness of metal augmentation was applied when necessary. Structural or bulk allografts were used in patients with severe bone defects. Two levels of constraint, PS and CCK prostheses, were applied; the levels of constraint were PS in 39 cases and CCK prostheses in 2 cases.

Isometric exercises using the extensor and flexor muscles were initiated shortly after surgery. The drain was removed on the second postoperative day, followed by the initiation of active and assisted range of motion (ROM) exercise. Full weight-bearing ambulation was started at 4 days, to the extent that the patient’s condition permitted. Mechanical prophylaxis for postoperative deep vein thrombosis (DVT)/pulmonary thromboembolism (PTE), including compression stocking or pneumatic compression device, was applied from the second postoperative day.

### Clinical evaluation

Clinical data were recorded before the operation and at the final outpatient department (OPD) visit (Table 1). The Western Ontario and McMaster Universities Osteoarthritis Index (WOMAC) was used to assess pain and function [8]. The ROM was measured using a long-armed goniometer.

**Table 1:** Distribution of follow-up period to the final outpatient department (OPD) visit after primary and revision total knee arthroplasty (TKA) in octogenarians

Last follow up (≤ postoperative year)	Primary		Revision	
	Number	%	Number	%
1 Year	10	4.3	3	7.3
2 Years	97	42	14	34.1
5 Years	77	33.3	14	34.1
10 Years	38	16.5	9	22
15 Years	9	3.9	1	2.4

The distribution was not significantly different between primary and revision TKA groups in octogenarians (*p* = 0.751)

### Radiographic evaluation

Preoperative (just before primary or revision TKA) and postoperative (2 weeks after primary or revision TKA) standing position, anteroposterior (AP) and lateral projection radiographs and orthoroentgenograms (full-length, standing position, AP projection radiographs) were acquired to assess limb alignment and component positioning. The mechanical axis (MA) was defined as the angle between the femoral and tibial mechanical axes on the orthoroentgenograms [9]. The positions of all femoral and tibial components were measured as the α, β, γ, and δ angles using the Knee Society radiographic evaluation method [10].

### Postoperative complications

The incidence of postoperative complications was determined by reviewing our hospital database. All complications recorded in the database were presented.

Specific complications associated with TKA were evaluated. The incidence of aseptic or septic component failures, periprosthetic fracture, and DVT/PTE was investigated.

Systemic complications, defined as worsening of underlying systemic disease or aggravation of a new problem during follow up, were also evaluated. Adverse systemic events in the cardiac, pulmonary, nephrotic, gastrointestinal, hepatic, endocrine, cerebral, and urologic systems were investigated, as was the occurrence of postoperative delirium.

### Length of hospital stay, intensive care unit (ICU) admission, and 90-day readmission

Length of hospital stay was calculated based on the admission and discharge days. The incidence of ICU admission and readmission within 90 days after surgery were also investigated.

### Lifetime survival analysis

A lifetime survival analysis after surgery was conducted using the Kaplan–Meier method based on operation day, death date, or last lifetime survival day. For investigation of lifetime survival, the patients or their family were contacted by telephone or mail. If the patients were dead, the death date was checked with the patients’ families, with consent. If the patients survived, the contact day was determined as the last lifetime survival day. The lifetime survival rate was analyzed over the interval of one year.

### Statistical analyses

The preoperative and postoperative clinical and radiographic results were compared in each group (paired *t* test). The clinical and radiographic results and length of hospital stay were compared between the primary and revision groups (independent samples *t* test). The occurrence of postoperative complications, ICU admission, and 90-day readmission was compared between the two groups (chi-square or Fisher’s exact test). The log-rank test was used for comparing Kaplan–Meier survival curves between the two groups. The authors performed all statistical analyses using the Statistical Package for the Social Sciences ver. 20.0 (IBM Corp., Armonk, NY, USA), and *p* < 0.05 was taken to indicate statistical significance.

A power analysis was performed to determine the minimum sample size affording sufficient power, with lifetime survival rate as the primary outcome. The power and alpha level were set to 0.8 and 0.05, respectively. As a result, the appropriate sample sizes were 222 primary TKA cases and 40 revision TKA cases; it was determined that our sample size was adequately powered. Further post-hoc analysis was performed to evaluate the power for other variables of the postoperative outcomes. Power > 80% was considered sufficient, and all of the variables that were significant met the criterion.

## Results

### Demographics

The mean age was 81.9 years (standard deviation (SD) 2.2) in the primary group and 82.3 years (SD 2.2) in the revision group (*p* = 0.310). The American Society of Anesthesiologists (ASA) score was not different (2.3 (SD 0.4) vs. 2.4 (SD 0.5), *p* = 0.144), but the age-adjusted Charlson comorbidity index (ACCI) was significantly higher preoperatively in the revision group (4.4 (SD 0.6) vs. 4.8 (SD 0.8), *p* = 0.003) [11, 12]. There were no differences in demographics with regard to sex, operation side, or body mass index, or in the mean follow-up period to the final OPD visit (Table 2).

**Table 2:** Patient demographics in octogenarians who underwent primary or revision total knee arthroplasty

	Primary	Revision	*p* value
Knee	231	41	
Age	81.9 ± 2.2 (80–90)	82.3 ± 2.2 (80–87)	0.310
Female/male	212/19	37/4	0.761
Right/left	116/115	20/21	1.000
Body mass index (kg/m^2^)	25.0 ± 3.0 (17.8–33.8)	25.6 ± 5.6 (18.1–30.9)	0.318
ASA score	2.3 ± 0.4 (2–3)	2.4 ± 0.5 (2–3)	0.806
ACCI	4.4 ± 0.6 (4–7)	4.8 ± 0.8 (4–8)	0.003
Last follow-up period (year)^a^	3.8 ± 0.6 (0.5–13)	3.5 ± 3.0 (0.5–11)	0.451

Data on the age, body mass index, American Society of Anesthesiologists (ASA) score, age-adjusted Charlson comorbidity index (ACCI), and last follow-up period are presented as the mean ± standard deviation (range)

^a^Last follow-up period is the follow-up period up to the last outpatient department visit

### Clinical and radiographic results

The WOMAC score and ROM improved after surgery in both groups (*p* < 0.001, respectively). The postoperative WOMAC was better after primary TKA compared with revision TKA (33.1 (SD 11.2) vs. 47.2 (SD 21.1), *p* < 0.001). The preoperative and postoperative ROM were greater after primary TKA (Table 3).

**Table 3:** Comparison of clinical results between groups of octogenarians who underwent primary or revision total knee arthroplasty

		Primary	Revision	*p* value
WOMAC score	Preoperative	64.2 ± 11.2 (45–80)	61.2 ± 12.3 (44–85)	0.142
	Postoperative	33.1 ± 11.2 (11–58)	47.2 ± 21.1 (10–70)	< 0.001
Range of motion (degrees)	Preoperative	119.2 ± 19.9 (50–140)	104.9 ± 29.7 (60–140)	0.005
	Postoperative	128.9 ± 12.1 (110–145)	113.6 ± 24.1 (80–145)	< 0.001

Data are presented as the mean ± standard deviation (range)

*WOMAC* Western Ontario and McMaster Universities Osteoarthritis Index

The MA showed more preoperative varus alignment in primary TKA (Table 4). The postoperative MA was varus 1.0° (SD 3.3°) in primary TKA and varus 2.8° (SD 4.3°) in revision TKA (*p* = 0.003). The positions of the components were appropriate in both primary and revision TKAs (Table 4).

**Table 4:** Comparison of the radiographic results in octogenarians who underwent primary or revision total knee arthroplasty

		Primary	Revision	*p* value
Mechanical axis (degrees)	Preoperative	Varus 12.3 ± 7.3 (varus 27.3 – valgus 15.1)	Varus 8.8 ± 9.7 (varus 22.8 – valgus 9.8)	0.045
	Postoperative	Varus 1.0 ± 3.3 (varus 5.3 –valgus 4.7)	Varus 2.8 ± 4.3 (varus 7.4 – valgus 5.2)	0.003
Position of components (degrees)	α angle	95.6 ± 1.9 (92.6–99.8)	95.7 ± 2.9 (92.1–99.9)	0.938
	β angle	90.8 ± 2.0 (86.3–93.7)	90.5 ± 1.7 (86.6–93.9)	0.316
	γ angle	4.2 ± 3.3 (− 2.5–7.9)	3.2 ± 4.1 (− 2.3–7.2)	0.096
	δ angle	86.2 ± 3.1 (84.5–89.7)	86.1 ± 3.7 (81.5–90.2)	0.923

Data are presented as the mean ± standard deviation (range)

### Postoperative complications

There were no cases of aseptic or septic component failure in either group. One case of periprosthetic fracture was observed in the revision group (0% vs. 2.4%, *p* = 0.151); a non-displaced fracture occurred on the medial femoral condyle, and conservative treatment was performed. There were three cases of DVT/PTE (one case of DVT and two cases of PTE) in the primary TKA group (1.3% vs. 0%, *p* = 1.000); thrombolytic therapy was applied, and all of the thrombotic problems were resolved. The most common postoperative systemic complication in both groups was delirium (7.4% vs. 14.6%, not significantly different), and there were no differences in the other systemic complication rates between the two groups (Table 5).

**Table 5:** The incidence of postoperative complications after primary or revision total knee arthroplasty in octogenarians

	Primary	Revision	*p* value
Cardiac	4 (1.7%)	2 (4.9%)	0.224
Pulmonary	6 (2.6%)	3 (7.3%)	0.139
Nephrotic	2 (0.9%)	0 (0%)	1.000
Gastrointestinal, hepatic	3 (1.3%)	1 (2.4%)	0.482
Endocrinologic	0	1 (2.4%)	0.151
Cerebral	1 (0.4%)	1 (2.4%)	0.279
Delirium	17 (7.4%)	6 (14.6%)	0.131
Urologic	13 (5.6%)	1 (2.4%)	0.702

### Length of hospital stay, ICU admission, and 90-day readmission

The mean length of hospital stay was 10.7 days (SD 8.7 days) in the primary group and 16.0 days (SD 9.5 days) in the revision group (*p* < 0.001). Two patients were admitted to the ICU after primary TKA (0.9% vs. 0%, *p* = 1.000). The reasons for ICU admission were aggravation of chronic obstructive pulmonary disease and stable angina, respectively. Appropriate medical treatment was performed and both patients recovered well.

There were three cases of readmission within 90 days after surgery in the primary group (1.2% vs. 0%, *p* = 1.000). The reasons for readmission were aggravated chronic kidney disease, pneumonia, and colorectal cancer, respectively.

### Lifetime survival analysis

**Fig. 1:**
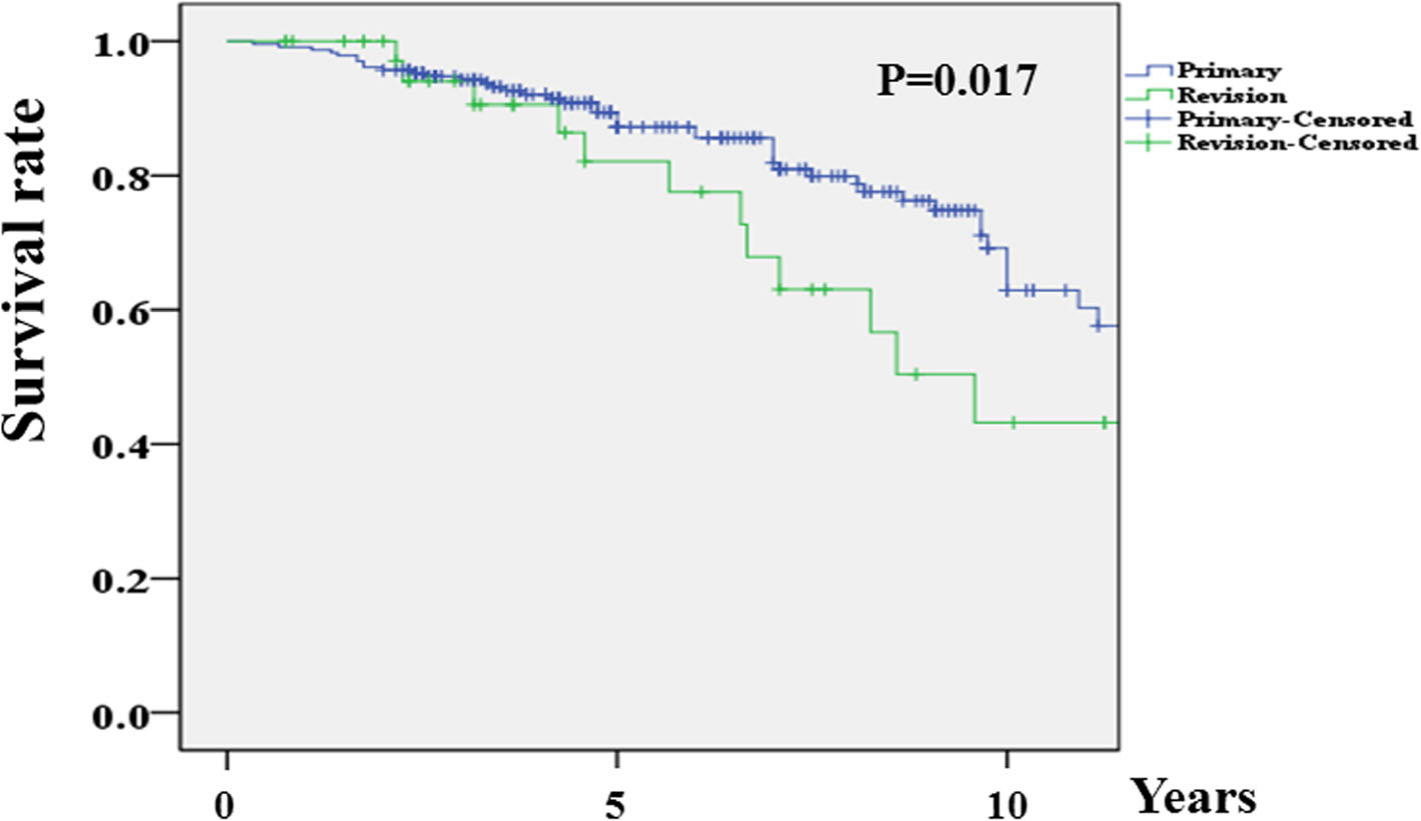
Lifetime survival curves (endpoint death) after primary or revision total knee arthroplasty in octogenarians

The 5-year and 10-year lifetime survival rates were 87.2% and 62.9%, respectively, in the primary group and 82.1% and 42.2%, respectively, in the revision group (*p* = 0.017) (Fig. 1).

## Discussion

The most important finding was that postoperative clinical results, TKA-related complication rates, and mid-term lifetime survival after primary and revision TKA in octogenarians were satisfactory. Additionally, there was no significant difference in overall postoperative complication rates between primary and revision TKA in octogenarians. The postoperative WOMAC score and ROM, length of hospital stay, and lifetime survival were better in the primary group than in the revision group.

Many previous studies have already reported satisfactory clinical outcomes and improved quality of life after primary TKA in octogenarians [3]. However, few studies have specifically investigated revision TKA in patients over 80 years old. Pagano et al. [4] reported satisfactory results after revision TKA in seven patients over the age of 90 years. One recent study compared 30-day perioperative complications, length of hospital stay, and readmission after revision TKA in octogenarians and two younger patient populations (< 70 and 70–79 years) [1]. They revealed only a higher rate of blood transfusion and slightly longer length of hospital stay in the octogenarians and suggested that octogenarians need not be discouraged from revision TKA based on their advanced age.

To our knowledge, the present study is the first to perform lifetime survival analysis with an endpoint set as death, after primary and revision TKA in octogenarians. In addition, our study is unique in that it compares the detailed postoperative outcomes, including clinical and radiographic results, incidence of postoperative complications, length of hospital stay, ICU admission rate, 90-day readmission rate, and lifetime survival rate after primary and revision TKA in octogenarians.

As with the previous studies [3], the present study showed improvement in clinical results after primary TKA in octogenarians. In revision TKA, the WOMAC score and ROM were also improved after the procedure, although they were worse than in primary TKA, as expected [6].

Postoperative life expectancy is an important factor to consider in determining surgery, especially in a vulnerable population. Lifetime survival is considered satisfactory when the 5-year survival rate is > 80% [13]. Yao et al. [14] reported the lifetime survival rate with the endpoint as death after revision TKA in a cohort with a mean age of 68.0 years; the 5-year lifetime survival rate after revision TKA due to aseptic failure was > 80%, and the rate after revision TKA due to periprosthetic joint infection was approximately 80%. In the present study in octogenarians, the 5-year lifetime survival rate was 87.2% after primary TKA and 82.1% after revision TKA with no prosthesis-related complication. There seems to be no reason to avoid primary or revision TKA because of concern about life expectancy in octogenarians.

A previous study showed that the mortality risk was significantly worse in patients undergoing revision TKA (risk ratio = 1.42) than in those undergoing primary TKA [14]. Similarly, the present study showed a significantly lower lifetime survival rate after revision TKA compared with primary TKA. One of the reasons for this might be related to a worse preoperative ACCI, which has been proven to be more appropriate and an excellent prognostic indicator of lifetime survival than other prognostic indexes [12, 15]. It seems that the ACCI can be considered as a tool for evaluating the risk-benefit when revision surgery is the only treatment option for the octogenarian with poor health due to comorbidity. Additionally, decreased function after revision TKA could be associated with greater risk of mortality [16].

There were no significant differences between primary and revision TKA in the rate of postoperative complications, ICU admission, or 90-day readmission in the present study. Although revision TKA is a challenging procedure, component removal is typically less arduous than revision hip arthroplasty, and the duration of surgery and blood loss are typically only slightly increased compared to those in primary TKA, except in cases with major bone loss or when a hinge prosthesis has to be used [6]. In addition, the rate of adverse events can be decreased when revision TKA is performed in a tertiary medical center by an experienced surgeon [17]. The only related index showing a significant difference between the two groups was length of hospital stay. This would be due to the need for more postoperative rehabilitation in the revision group considering their more limited ROM and poorer patient function [6].

In the present study, delirium was the most common complication in octogenarians after primary and revision TKA (7.4% vs. 14.6%, *p* = 0.131). Older age is known to be an important predisposing factor for delirium [18]. A systematic review reported 0–10% incidence of delirium after TKA in patients with an average age > 70 years [19]. Delirium needs to be managed appropriately as the most common systemic complication in both primary and revision TKA in octogenarians.

Interestingly, there was no difference between primary and revision TKA in the delta angle, which represents postoperative tibial slope, although the primary group included CR TKA in which the target of the posterior slope was different from that in PS and CCK TKA [20]. This could be because the tibial slope was adjusted to obtain appropriate soft tissue balance in specific surgical situations [21]. The soft tissue tension, including the posterior cruciate ligament in octogenarians may have been lax, but it is difficult to draw meaningful conclusions from a retrospective study.

As limited resources exert pressure on our healthcare system, assessing the rationality of certain procedures becomes an important issue. Investigating lifetime survival and incidence of complications after procedures in older patients will help to assess cost and risk benefit, which are important factors of rationality. The satisfactory clinical results, TKA-related complication rate, and mid-term lifetime survival in the present study seem to justify primary and revision TKA in octogenarians. Considering the similar overall complication rates, revision TKA need not be avoided more than primary TKA in octogenarians.

The present study had several limitations. First, this was a retrospective study, which can lead to inaccuracies in the incidence of complications or death date. Second, the number of patients who underwent revision TKA was small due to the fact that the data came from a single institute and the operating period began in 2000. Further study in a large cohort is required. Third, the study cohort was from a tertiary care institution in an Asian country. Our results may not be representative of primary and revision TKA in octogenarians in other countries and hospitals. Fourth, the follow-up period to the final OPD visit was short. A large proportion of patients (46.3% of those undergoing primary TKA and 41.4% of those undergoing revision TKA) were followed for 2 years or less. This could be the reason for the low incidence of periprosthetic failure. However, we contacted subjects by telephone or mail to analyze lifetime survival. Fifth, we were unable to evaluate the functional results just before the death of patients. The clinical results of the last follow up were evaluated in our hospital database, but the date of death was investigated by telephone or mail communication with the patients’ families. In addition, there was lack of information about the cause of death because it was reported by family members. Sixth, the estimated blood loss and transfusion rate were not investigated; such information could be useful for developing blood management strategies for primary or revision TKA in octogenarian patients. Seventh, we could not compare our lifetime survival curve with that of the general octogenarian population in our country. Because this is a single-institute study, data on the general population could not be accessed. Lifetime survival of octogenarians after primary or revision TKA could have been studied more objectively if this comparison could be made. Last, we could not investigate if patients were discharged home or to a rehabilitation center because there was no information about this in our hospital database. This information may have helped assess the socioeconomic costs after primary and revision TKA in octogenarians. Further study will be required to obtain this relevant information.

## Conclusion

Both primary and revision TKA are viable options for octogenarians, based on the satisfactory clinical outcomes, TKA- related complication rates, and mid-term lifetime survival. Delirium needs to be managed appropriately as the most common systemic complication in both primary and revision TKA in octogenarians.

## Data Availability

The datasets generated and/or analyzed during the current study are not publicly available, but they are available from the corresponding author on reasonable request.
